# Preclinical evaluation of a live attenuated bivalent Trichophyton vaccine for prophylaxis and post-infection treatment of dermatophytosis in rabbits

**DOI:** 10.14202/vetworld.2026.4143-4158

**Published:** 2026-09-18

**Authors:** Mynbay Umitzhanov, Nurbol Bakirov, Abylay Sansyzbay, Ainur Nurpeisova, Nazym Akimzhan, Begali Kanatov, Amangeldy Maulanov, Serik Kanatbayev, Malik Yussupov, Markhabat Kassenov, Abay Zhandos, Nurbek Aldayarov, Zhibek Zhetpisbay, Raikhan Boranbaeva, Gulnara Abilkassimova, Sholpan Iglikova, Altynai Arysbekova

**Affiliations:** 1 Department of Biological Safety, Kazakh National Agrarian Research University, Almaty, Republic of Kazakhstan.; 2 Kazakh Scientific Research Veterinary Institute LLP, Almaty, Republic of Kazakhstan.; 3 Department of Biology, Faculty of Sciences, Kyrgyz-Turkish Manas University, Bishkek, Kyrgyz Republic.; 4 Department of Computer Science, Al-Farabi Kazakh National University, Almaty, Republic of Kazakhstan.; 5 Vetinvest Innovative Enterprise LLP, Erkin, Talgar District, Almaty Region, Republic of Kazakhstan.; 6 Scientific and Practical Center for Dermatovenereology and Cosmetology, Kazakh-Russian University, Almaty, Republic of Kazakhstan.; 7 Kazakh-Russian Medical University, Almaty, Republic of Kazakhstan.

**Keywords:** bivalent vaccine, bovine trichophytosis, dermatophytosis, live attenuated vaccine, One Health, rabbit model, Trichophyton mentagrophytes, Trichophyton verrucosum

## Abstract

**Background and Aim:**

Bovine trichophytosis is a contagious zoonotic dermatophytosis that adversely affects animal health and livestock productivity and poses an occupational risk to people in contact with infected cattle. Although live attenuated vaccines have contributed to disease control, evidence for bivalent formulations targeting multiple *Trichophyton* species remains limited. This study evaluated the prophylactic activity and post-infection outcomes of a live attenuated bivalent vaccine candidate containing *Trichophyton verrucosum* and a member of the *Trichophyton mentagrophytes* species complex in an experimental rabbit model.

**Materials and Methods:**

Thirty-three clinically healthy rabbits were allocated to a prophylactic group (Group I, n = 10), therapeutic group (Group IIa, n = 10), infected untreated histopathology subgroup (Group IIb, n = 3), and intact control group (Group III, n = 10). Group I received two intramuscular vaccinations 14 days apart and was challenged 21 days after the second vaccination, whereas Group IIa was experimentally infected before therapeutic vaccination. Clinical manifestations, body weight, rectal temperature, hematological parameters, mycological culture, and histopathological changes were evaluated. Clinical and histopathological assessments were descriptive and non-blinded.

**Results:**

Following experimental challenge, no clinically visible dermatophytosis lesions occurred in prophylactically vaccinated rabbits (0/10), whereas all infected untreated reference rabbits developed lesions (3/3; two-sided Fisher’s exact test, p = 0.0035), supporting prophylactic activity. Therapeutically vaccinated rabbits showed progressive regression of visible crusting and hair regrowth; however, the absence of a longitudinal untreated infected group and positive treatment comparator precluded attribution of recovery specifically to vaccination. Dermatophyte growth was recovered from challenged skin after 5–7 days, although species-specific identification and quantitative fungal burden were not determined. Hematological indices showed limited descriptive variation. Histopathology demonstrated inflammatory and degenerative cutaneous lesions in untreated infected rabbits and nonspecific reactive lymphoid changes in vaccinated animals.

**Conclusion:**

The live attenuated bivalent vaccine candidate demonstrated promising prophylactic activity against experimental dermatophytosis in rabbits. Post-infection clinical improvement provides preliminary evidence warranting further investigation but cannot establish therapeutic efficacy. Controlled studies in the target host should incorporate adequate comparator groups, standardized lesion scoring, quantitative and species-specific mycology, immunological endpoints, and comprehensive vaccine quality control and stability assessments before field application.

## INTRODUCTION

Bovine dermatophytosis, commonly termed ringworm or trichophytosis, is a contagious fungal skin disease caused principally by *Trichophyton verrucosum*. Transmission occurs through direct contact between animals or indirectly through contaminated hair, skin scales, housing surfaces, equipment, and other fomites. Young cattle are particularly susceptible, and outbreaks may impair growth, reduce hide quality, increase treatment and disinfection costs, and complicate animal movement and herd management [1–5]. The disease also has an important One Health dimension because zoophilic dermatophytes can be transmitted from cattle to farmers, veterinarians, animal handlers, farm workers, and household contacts. Farm-level investigations have documented *T. verrucosum* infection in cattle and exposed humans, while mixed rearing of cattle with other ruminants has been associated with a higher occurrence of human infection [4, 6, 7]. Therefore, occupational prevention requires coordinated veterinary and public health measures, including early identification and isolation of infected cattle, use of protective clothing and gloves, environmental cleaning, disinfection of shared equipment, and appropriate medical referral of exposed individuals with compatible skin lesions.

Published epidemiological data on bovine trichophytosis in Kazakhstan and Central Asia remain scarce. A recent study in Kazakhstan characterized 12 cattle-associated *Trichophyton* isolates collected from six regions and established the first regional molecular and mitochondrial genome baseline for *T. verrucosum*-related isolates; however, that investigation was not designed as a prevalence survey [8]. Nevertheless, recovery of isolates from geographically separated regions confirms the circulation of cattle-associated dermatophytes across different livestock-production areas of Kazakhstan and provides a basis for future epidemiological monitoring. Under intensive cattle-management conditions, close animal contact, contaminated hair and skin scales, shared equipment, increased humidity, inadequate ventilation, and persistence of fungal elements in the production environment may facilitate transmission. In adjacent Ningxia, China, clinical dermatophytosis was detected in 74 of 482 examined cattle (15.35%), with occurrence varying according to locality, season, and age category [2]. Although these findings cannot be directly extrapolated to Kazakhstan, they indicate the potential importance of environmental and management-related factors in regional cattle-production systems. Systematic surveillance in Kazakhstan should therefore integrate standardized clinical examination, mycological culture, and molecular identification [9, 10] to determine animal- and herd-level prevalence, characterize circulating dermatophyte species and strains, and identify age- and management-related risk factors.

Although *T. verrucosum* is the principal etiological agent of bovine ringworm, members of the *Trichophyton mentagrophytes* species complex and other dermatophytes have also been recovered from cattle and farm environments [2, 3, 9, 11]. The epidemiological contribution of the *T. mentagrophytes* species complex in Kazakhstan remains insufficiently defined. Consequently, its inclusion in the present vaccine formulation was based on broader veterinary evidence and the hypothesis that a bivalent formulation could provide wider antigenic coverage rather than on a demonstrated estimate of its local prevalence. Vaccination has contributed to the long-term control of bovine ringworm in several countries, although most established vaccines for cattle are monovalent live attenuated *T. verrucosum* preparations [12–14]. Researchers have also evaluated experimental monovalent, bivalent, and polyvalent dermatophyte vaccines in cattle, rabbits, and guinea pigs [15–17]. Rabbit models provide a controlled platform for preliminary evaluation of cutaneous challenge, lesion development, and tissue responses before studies in the target host; however, findings obtained in rabbits cannot establish vaccine efficacy in cattle [17, 18].

Despite previous evaluations of monovalent, bivalent, and polyvalent dermatophyte vaccines, including prophylactic and post-infection administration in laboratory animal models [15–18], several important knowledge gaps remain. In particular, published evidence is limited for live attenuated formulations specifically combining *T. verrucosum* with a member of the *T. mentagrophytes* species complex. Evidence is also limited on evaluating both prophylactic activity and clinical outcomes after post-infection administration of such a combined formulation within the same experimental design. Furthermore, the relative contribution of individual dermatophyte components within combined formulations remains insufficiently characterized. Interpretation of post-infection vaccination is particularly challenging because clinical improvement cannot be attributed to vaccination without appropriate longitudinal untreated and positive treatment comparator groups. Thus, exploratory preclinical evaluation is required to characterize the biological and clinical responses associated with this specific bivalent formulation before controlled studies are undertaken in the target host. To our knowledge, this is the first published preclinical evaluation of this specific live attenuated combined formulation incorporating both prophylactic and post-infection experimental arms within the same rabbit model.

Therefore, this study aimed to obtain exploratory preclinical evidence on a live attenuated bivalent *Trichophyton* vaccine candidate containing *T. verrucosum* and a member of the *T. mentagrophytes* species complex using an experimental rabbit model. Specifically, the study evaluated prophylactic activity following vaccination and subsequent experimental challenge, clinical outcomes following post-infection vaccine administration, hematological tolerability during the experimental period, and histopathological changes associated with infection and vaccination. The study was designed to provide preliminary evidence to support further evaluation of the candidate formulation, rather than to establish definitive therapeutic efficacy or efficacy in cattle.

## MATERIALS AND METHODS

### Ethical approval

All experimental procedures involving rabbits were conducted in accordance with applicable national and international requirements for the ethical use of animals in scientific research, including the UK Animals (Scientific Procedures) Act 1986 and Directive 2010/63/EU of the European Parliament and of the Council on the protection of animals used for scientific purposes. The experimental protocol, including animal housing and handling, vaccination, experimental infection, clinical monitoring, blood collection, and scheduled euthanasia and tissue collection, was reviewed and approved by the Ethics Committee for Animal Experiments of the Kazakh National Agrarian Research University (Protocol No. 8, April 1, 2023). All procedures were performed in accordance with the approved experimental protocol, with appropriate measures taken to minimize animal discomfort and unnecessary suffering.

### Study period and location

The experimental study was conducted from March 2025 to April 2026 at the laboratory of the Kazakh Scientific Research Veterinary Institute LLP (KazSRVI), Almaty, Kazakhstan. The study included preparation of the live attenuated vaccine candidate; evaluation of its prophylactic activity and clinical outcomes following post-infection administration in rabbits; clinical monitoring; hematological assessment; mycological examination; and histopathological evaluation.

### Vaccine strains, attenuation, and preparation

The vaccine candidate was prepared from production cultures of *T. verrucosum* and a phenotypically identified member of the *T. mentagrophytes* species complex. The production cultures had previously undergone seven serial passages on wort agar during strain development to reduce virulence while retaining the cultural characteristics required for vaccine preparation. Serial passaging was used as the attenuation procedure during strain development. This study did not evaluate genomic stability, comparative virulence relative to the original isolates, or formal reversion-to-virulence testing.

The two cultures were propagated separately on wort agar under stationary conditions at 28 ± 1°C. Fungal biomass was harvested aseptically and suspended in sterile distilled water. Microconidia were counted using a Goryaev chamber. The two suspensions were subsequently combined at a 1:1 ratio, and the final concentration was adjusted to 2.0 × 10⁷ microconidia/mL. Gelatin and sucrose were added at final concentrations of 4% (w/v) each before lyophilization [16, 17]. Dedicated sterility testing of the final lyophilized preparation, quantitative assessment of post-lyophilization viability, genetic stability testing, batch-to-batch consistency assessment, and long-term stability testing were not performed.

### Challenge inoculum and experimental infection

Experimental infection was performed using an 18–20-day-old virulent homologous *Trichophyton* culture preparation containing *T. verrucosum* and a member of the *T. mentagrophytes* species complex. The final challenge dose was standardized to 7 × 10⁶ fungal elements per animal. The relative concentration of each species within the challenge preparation was not quantified separately.

A 5 × 5 cm area of the dorsal region was clipped and treated with 70% ethanol. The skin was then scarified using a sterile blade until slight bleeding was visible, after which the challenge culture was applied by gentle rubbing. No occlusive dressing was used. The rabbits received routine husbandry and were observed daily after experimental challenge [18, 19].

Pathological material was collected from the challenged skin and inoculated onto wort agar. Characteristic dermatophyte growth was observed after 5–7 days. The recovered cultures were not quantified as colony-forming units (CFU)/cm² and were not subjected to species-specific polymerase chain reaction (PCR) or sequencing. Therefore, simultaneous infection with both dermatophyte components and their relative contributions to the experimental infection were not independently confirmed.

### Animals, housing, randomization, and blinding

Thirty-three clinically healthy outbred rabbits of both sexes, 4–5 months of age and weighing 2.5–3.0 kg, were obtained from specialized breeding facilities in Kazakhstan. The rabbits were housed individually in ventilated cages under standard laboratory conditions and monitored daily for general health, behavior, feed and water intake, skin condition, and clinical abnormalities.

Following acclimatization and confirmation of clinical health, the rabbits were randomly allocated to four experimental groups. The exact method used to generate the random allocation sequence was not retained in the records available for this revision, and allocation concealment was not performed. The same investigators conducted clinical observations throughout the experiment and were aware of group allocation. Histopathological assessments were also performed without blinding.

### Study design

The study comprised four experimental arms designed to evaluate prophylactic activity, clinical outcomes following post-infection vaccination, untreated early histopathological changes, and physiological and histological findings in intact controls. Figure 1 shows a schematic of the experimental design and sampling schedule. Group I (prophylactic group, n = 10) received vaccination before experimental challenge and was used to evaluate prophylactic activity. Group IIa (therapeutic group, n = 10) was experimentally infected before vaccination and was used to evaluate clinical outcomes following post-infection vaccine administration. Group IIb (infected untreated histopathology subgroup, n = 3) was included to provide an early histopathological reference for untreated dermatophytosis. Group III (intact control group, n = 10) comprised rabbits that were neither infected nor vaccinated and served as physiological and histological controls.

The experimental protocol prespecified a prophylactic dose of 2.0 mL and a post-infection dose of 4.0 mL. The post-infection regimen used a twofold greater administered volume as an exploratory approach after establishment of experimental infection. Because no dose-ranging comparison was performed, these administered volumes should not be interpreted as either the minimum effective prophylactic dose or the optimized post-infection dose.

**Figure 1:**
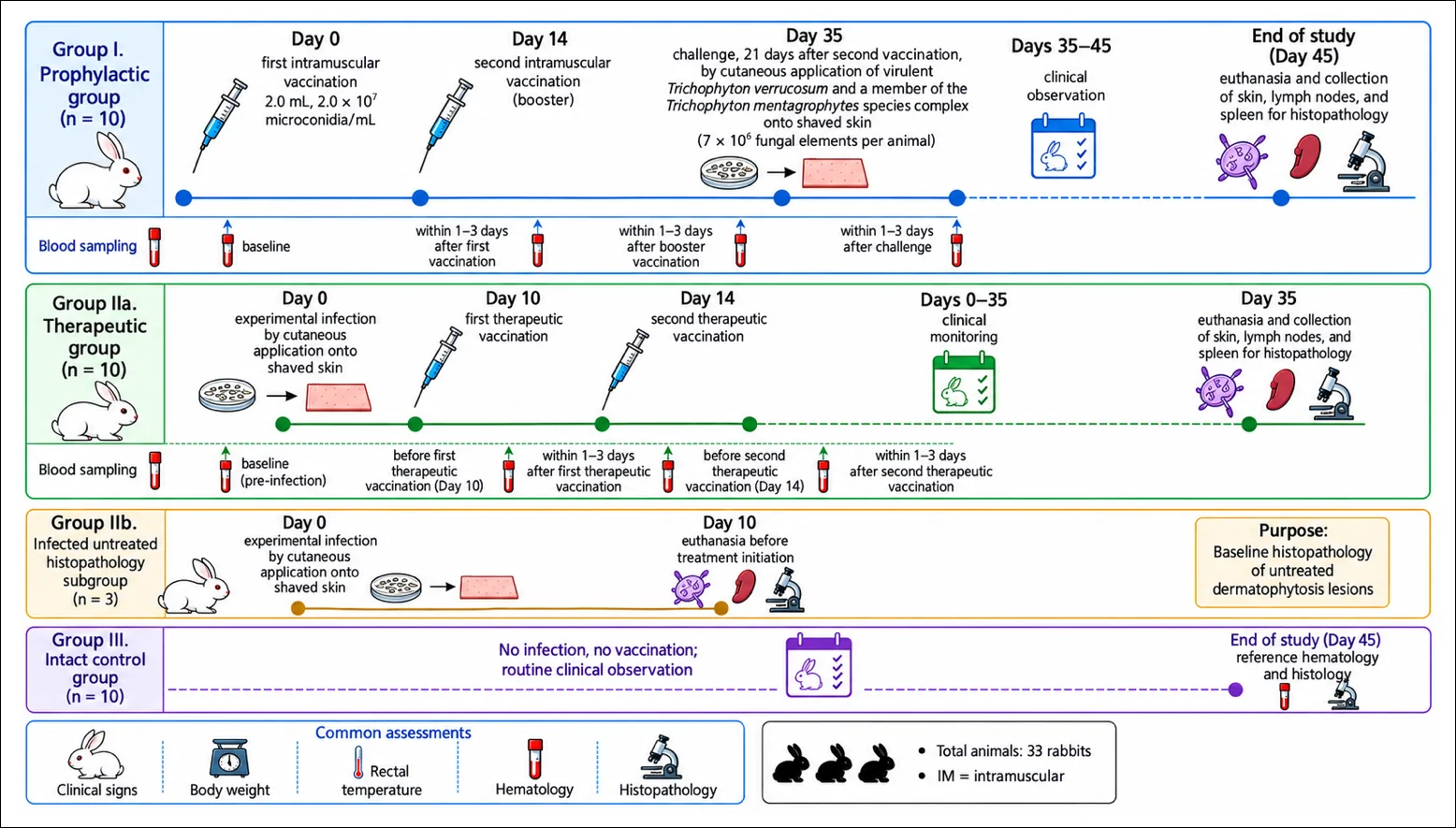
Study design and sampling schedule. Group I (n = 10) received two prophylactic vaccinations 14 days apart and was challenged 21 days after the second vaccination. Group IIa (n = 10) was infected before therapeutic vaccination. Group IIb (n = 3) was infected and euthanized on day 10 as an early untreated histopathological reference. Group III (n = 10) was neither infected nor vaccinated.

### Clinical monitoring

Rabbits were examined daily for general condition, behavior, appetite, water intake, coat condition, erythema, scaling, alopecia, crust formation, lesion extension, and hair regrowth. Body weight and rectal temperature were recorded according to the predefined study schedule. Skin lesions were assessed descriptively because the study did not use a validated numerical or semiquantitative lesion-severity scoring system.

### Blood collection and hematological analysis

Blood samples were collected into ethylenediaminetetraacetic acid (EDTA)-containing tubes at baseline and at the scheduled post-vaccination, post-revaccination, and post-challenge time points. Hematological parameters were measured using a Sysmex XP-100 automated hematology analyzer (Sysmex, Kobe, Japan). The evaluated parameters included white blood cell count (WBC), lymphocyte count (LYM), monocyte/intermediate cell count (Mon#/Mid), granulocyte count (GRA), red blood cell count (RBC), hemoglobin (HGB), hematocrit (HCT), mean corpuscular volume (MCV), mean corpuscular hemoglobin (MCH), mean corpuscular hemoglobin concentration (MCHC), red blood cell distribution width–coefficient of variation (RDW-CV), platelet count (PLT), mean platelet volume (MPV), platelet distribution width (PDW), and plateletcrit (PCT). Study-specific reference intervals were not established; therefore, the recorded values were not classified as normal or abnormal relative to a validated rabbit reference population.

### Euthanasia and tissue collection

All rabbits were euthanized at their scheduled experimental endpoints using a CO₂ euthanasia system (EVTANAYZER-2M, AWTech, Russia) in accordance with the approved animal experimentation protocol. Following confirmation of death, samples of skin, regional lymph nodes, and spleen were collected for histopathological examination. No animals required unscheduled euthanasia before the predefined experimental endpoints.

### Histopathological examination

Samples of skin, regional lymph nodes, and spleen were fixed in 10% neutral-buffered formalin for at least 24 h, embedded in paraffin, sectioned at a thickness of 5 μm, and stained with hematoxylin and eosin. Histological sections were examined for epidermal damage, hyperkeratosis, inflammatory infiltration, edema, vascular changes, necrosis, degeneration, and reactive lymphoid changes. Periodic acid–Schiff and Grocott–Gomori methenamine silver staining were not performed. Histopathological assessment was qualitative; no predefined grading system, blinded scoring procedure, or morphometric analysis was used.

### Statistical analysis

Statistical analyses were performed using Statistical analyses were performed using R software version 4.4.0 (R Foundation for Statistical Computing, Vienna, Austria; https://www.r-project.org/) with the tidyverse (https:// CRAN.R-project.org/package=tidyverse), lme4 (https://CRAN.R-project.org/package=lme4), lmerTest (https:// CRAN.R-project.org/package=lmerTest), emmeans (https://CRAN.R-project.org/package=emmeans), and DHARMa (https://CRAN.R-project.org/package=DHARMa) packages. Body weight and rectal temperature were analyzed using linear mixed-effects models. Experimental group, day, and the group × day interaction were included as fixed effects, whereas individual animal was included as a random intercept. Day was treated as a categorical variable to account for potentially nonlinear temporal changes.

The significance of fixed effects was assessed using Type III analysis of variance with Satterthwaite’s approximation for degrees of freedom. When a significant group × day interaction was identified, pairwise comparisons among groups were performed at predefined key time points (days 0, 14, 21, and 35) using Tukey’s adjustment for multiple comparisons. Model assumptions were assessed by visual inspection of residual plots and quantile–quantile plots and by examination of simulated residuals generated using the DHARMa package.

The incidence of clinically visible dermatophytosis lesions in the prophylactic group and the infected untreated histopathology reference group was compared using a two-sided Fisher’s exact test because of the small group sizes and zero cell frequencies.

Hematological parameters were summarized descriptively as group means with the reported measures of dispersion at each sampling time point. Formal longitudinal or between-group hypothesis testing was not performed for hematological outcomes because complete individual-level repeated measurements were unavailable for reliable mixed-effects or repeated-measures analysis.

No formal *a priori* power calculation was performed. The exploratory group size of 10 animals was selected with reference to group sizes used in comparable repeated-measures rabbit vaccine studies and animal welfare considerations [20, 21]. Statistical significance was set at p < 0.05.

## RESULTS

### Cultural and microscopic characteristics of attenuated vaccine cultures

The attenuated cultures of *T. verrucosum* and *T. mentagrophytes* retained characteristic macroscopic and microscopic features following serial passage on wort agar. After incubation at 28°C for 21 days, both cultures produced visible fungal growth suitable for vaccine preparation. Macroscopic examination demonstrated distinct colony morphologies for the two vaccine strains, whereas microscopic examination revealed fungal hyphae and conidial structures characteristic of *Trichophyton* spp. These findings showed that the production strains retained recognizable cultural and microscopic characteristics after serial passage and cultivation on wort agar (Figure 2).

**Figure 2:**
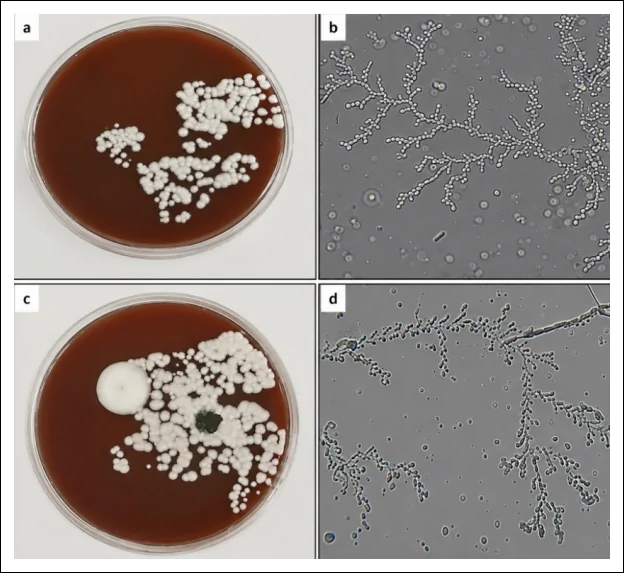
Cultural and microscopic characteristics of attenuated vaccine cultures of *Trichophyton verrucosum* and *Trichophyton mentagrophytes*. (a) Colony morphology of attenuated *T. verrucosum* on wort agar. (b) Microscopic features of attenuated *T. verrucosum* culture. (c) Colony morphology of attenuated *T. mentagrophytes* on wort agar. (d) Microscopic features of attenuated *T. mentagrophytes* culture. Magnification = ×100.

### Mycological recovery after experimental challenge

Pathological material collected from the challenged skin was cultured on wort agar. Characteristic dermatophyte growth was observed after 5–7 days, supporting the presence of viable fungal infection after experimental challenge. Species-specific identification of the recovered cultures was not performed; therefore, the relative contributions of the two dermatophyte components could not be determined.

### Clinical outcomes after prophylactic and post-infection vaccination

No clinically visible dermatophytosis lesions were recorded in the prophylactically vaccinated group following challenge (0/10), whereas all rabbits in the infected untreated histopathological reference group developed visible lesions by day 10 (3/3). The difference in lesion incidence was statistically significant according to the two-sided Fisher’s exact test (p = 0.0035; Table 1). Because Group IIb included only three rabbits and was euthanized during the early phase of infection, this comparison should be interpreted as supportive evidence of prophylactic activity rather than as a definitive estimate of vaccine efficacy.

**Table 1:** Clinical outcomes in rabbit groups following experimental dermatophyte infection or challenge.

**Group**	**Experimental role**	**Animals with clinically visible dermatophytosis lesions**	**Mortality**
Group I (n = 10)	Prophylactic vaccination followed by experimental challenge	0/10 after challenge	0/10
Group IIa (n = 10)	Experimental infection followed by therapeutic vaccination	10/10 after infection and before therapeutic vaccination; visible lesion regression and hair regrowth were observed during follow-up	0/10
Group IIb (n = 3)	Infected untreated early histopathological reference group	3/3 by day 10 post-infection	0/3 before scheduled euthanasia
Group III (n = 10)	Intact, non-infected, non-vaccinated control	0/10	0/10

Group IIb was euthanized on day 10 and served only as an early untreated histopathological reference group. It was not used as a longitudinal control for evaluating spontaneous recovery or therapeutic efficacy.

Rabbits in Group I remained clinically normal throughout the post-challenge observation period. No visible dermatophytosis lesions, alopecia, scaling, crust formation, or progressive skin lesions were recorded, and no mortality occurred (Figure 3a).

Rabbits in Group IIa developed visible signs of dermatophytosis after experimental infection and before therapeutic vaccination. Early clinical findings included localized erythema, edema, crust formation, and reduced activity in several animals. Following therapeutic vaccination, visible skin lesions gradually regressed, with reduced crusting and progressive hair regrowth during follow-up (Figure 3b). No mortality occurred. However, because no infected untreated group was followed for the same duration and no positive therapeutic comparator was included, the rate and cause of clinical recovery could not be determined.

Rabbits in Group IIb developed characteristic dermatophytosis lesions by day 10, including erythematous skin areas, crust formation, alopecia, and granulation tissue. These rabbits served as an early pathological reference for untreated infection (Figure 3c). Rabbits in Group III remained clinically normal throughout the observation period.

**Figure 3:**
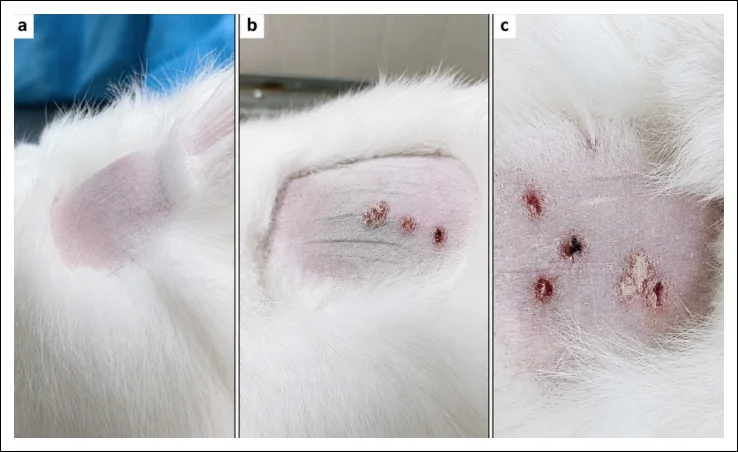
Clinical manifestations of experimental dermatophytosis in rabbits. (a) Prophylactic group vaccinated before challenge, showing no visible dermatophytosis lesions. (b) Therapeutic group after experimental infection and vaccination, showing regression of visible dermatophytosis lesions during recovery. (c) Infected untreated histopathological subgroup, showing typical dermatophytosis lesions characterized by erythematous skin areas, crust formation, and granulation tissue.

### Body weight and rectal temperature dynamics

Linear mixed-effects analysis identified significant effects of experimental group, day, and the group × day interaction on absolute body weight (group: F(2, 1370) = 1199.86, p < 0.0001; day: F(45, 1370) = 12.39, p < 0.0001; group × day: F(90, 1370) = 6.59, p < 0.0001; Figures 4A, C, and E).

The prophylactic group had a higher absolute body weight than the therapeutic and intact control groups at baseline. Consequently, between-group differences in absolute body weight were interpreted cautiously and were not attributed solely to vaccination. Following experimental infection, the therapeutic group showed a reduction in body weight followed by partial recovery during the observation period. The prophylactic group showed comparatively limited temporal variation after challenge, whereas the intact control group also demonstrated changes in body weight relative to baseline (Figures 4A, C, and E).

Rectal temperature showed a significant group × day interaction (F(88, 1340) = 42.25, p < 0.0001), indicating that temperature patterns differed among experimental groups over time (Figures 4B, D, and F). The therapeutic group showed transient elevations in rectal temperature during the acute post-infection period, followed by a gradual return toward baseline values. The prophylactic group showed no sustained elevation in rectal temperature after challenge. By the end of the observation period, rectal temperature values in the therapeutic group approached those recorded in the intact control group (Figures 4B, D, and F).

### Hematological observations

Group means of hematological values at the recorded sampling time points are presented in Tables 2 and 3. The summarized leukocyte, erythrocyte, and platelet indices showed relatively limited descriptive variation during the observation period. The available group-level values showed no consistent directional pattern indicative of a major hematological disturbance.

However, the hematological dataset did not contain complete individual-level repeated measurements suitable for reliable longitudinal or between-group inferential analysis. Therefore, the hematological findings are presented descriptively and cannot be interpreted as statistical confirmation of hematological safety or equivalence among experimental groups.

**Figure 4:**
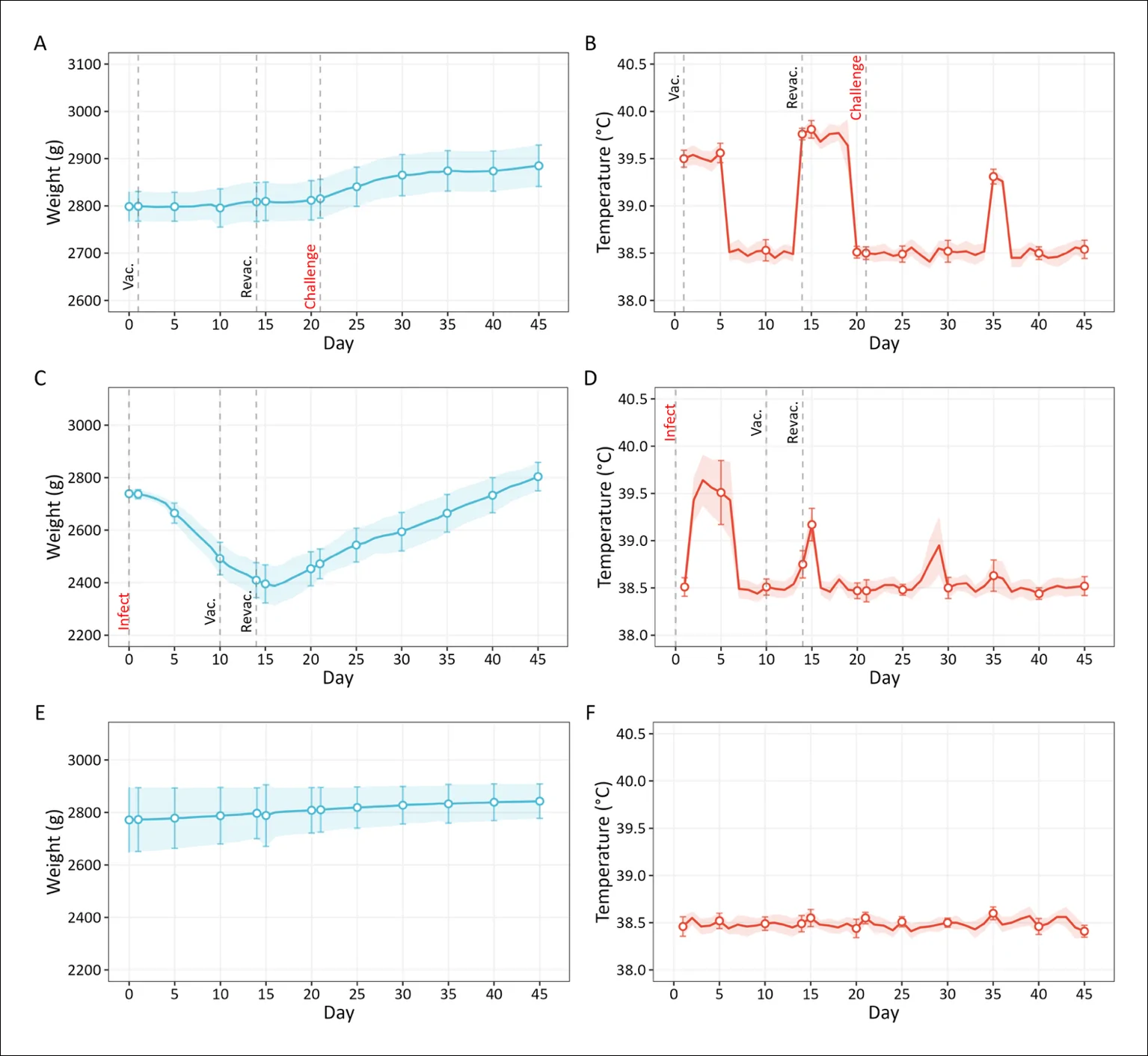
Changes in body weight and rectal temperature of rabbits during the experimental period. Body weight dynamics are shown for the prophylactic group (A), therapeutic group (C), and intact control group (E). Rectal temperature dynamics are shown for the prophylactic group (B), therapeutic group (D), and intact control group (F). Data are presented as group means with 95% confidence intervals. Body weight and rectal temperature were analyzed using linear mixed-effects models with group, day, and the group × day interaction as fixed effects and individual animal as a random intercept.

**Table 2:** Descriptive changes in hematological indices of rabbits in the prophylactic group (Group I, n = 10) at baseline and after the first vaccination, second vaccination, and experimental challenge.

**Hematological parameter**	**Baseline, day 0**	**First vaccination, day 1**	**First vaccination, day 2**	**First vaccination, day 3**	**Second vaccination, day 1**	**Second vaccination, day 2**	**Second vaccination, day 3**	**Challenge, day 1**	**Challenge, day 2**	**Challenge, day 3**
WBC (×10⁹/L)	11.2 ± 0.3	11.4 ± 0.3	11.8 ± 0.3	11.6 ± 0.2	11.4 ± 0.3	11.9 ± 0.3	11.7 ± 0.2	11.2 ± 0.2	11.5 ± 0.2	11.5 ± 0.2
LYM (×10⁹/L)	8.9 ± 0.2	9.0 ± 0.2	9.3 ± 0.2	9.2 ± 0.2	9.0 ± 0.2	9.3 ± 0.2	9.2 ± 0.1	9.1 ± 0.1	9.2 ± 0.1	9.1 ± 0.1
MID/Mon# (×10⁹/L)	0.4 ± 0.1	0.5 ± 0.1	0.6 ± 0.1	0.5 ± 0.1	0.5 ± 0.1	0.6 ± 0.1	0.5 ± 0.1	0.5 ± 0.1	0.6 ± 0.1	0.5 ± 0.1
GRA (×10⁹/L)	2.7 ± 0.1	2.6 ± 0.2	2.9 ± 0.1	2.8 ± 0.1	2.6 ± 0.2	2.9 ± 0.1	2.8 ± 0.1	2.9 ± 0.1	2.8 ± 0.1	2.9 ± 0.1
RBC (×10¹²/L)	8.9 ± 0.2	8.8 ± 0.2	9.2 ± 0.2	9.0 ± 0.2	9.1 ± 0.2	9.2 ± 0.2	9.0 ± 0.1	9.1 ± 0.1	9.0 ± 0.1	9.1 ± 0.2
HGB (g/L)	161 ± 2	160 ± 2	165 ± 2	163 ± 2	163 ± 2	164 ± 2	163 ± 2	163 ± 2	164 ± 2	163 ± 2
HCT (%)	49 ± 1	48 ± 1	52 ± 1	50 ± 1	51 ± 1	52 ± 1	50 ± 1	51 ± 1	50 ± 1	51 ± 1
MCV (fL)	69.5 ± 0.5	69.0 ± 0.5	70.5 ± 0.5	70.1 ± 0.3	70.0 ± 0.5	70.5 ± 0.5	70.2 ± 0.5	70.2 ± 0.5	70.1 ± 0.3	70.2 ± 0.5
MCH (pg)	23.3 ± 0.3	23.5 ± 0.3	23.6 ± 0.3	23.7 ± 0.3	23.5 ± 0.3	23.6 ± 0.3	23.5 ± 0.2	23.5 ± 0.3	23.5 ± 0.3	23.5 ± 0.3
MCHC (g/L)	378 ± 3	380 ± 3	382 ± 3	381 ± 3	382 ± 3	381 ± 3	381 ± 3	380 ± 3	380 ± 3	381 ± 3
RDW-CV (%)	15.8 ± 0.3	15.7 ± 0.3	16.2 ± 0.3	16.0 ± 0.3	16.3 ± 0.3	16.1 ± 0.3	16.0 ± 0.3	16.0 ± 0.3	16.1 ± 0.3	16.0 ± 0.3
PLT (×10⁹/L)	635 ± 10	630 ± 10	655 ± 10	645 ± 8	650 ± 10	645 ± 8	640 ± 8	645 ± 10	640 ± 8	640 ± 8
MPV (fL)	4.6 ± 0.2	4.5 ± 0.2	4.9 ± 0.2	4.8 ± 0.1	4.9 ± 0.2	4.8 ± 0.2	4.7 ± 0.1	4.7 ± 0.2	4.7 ± 0.2	4.7 ± 0.2
PDW (%)	15.4 ± 0.3	15.7 ± 0.3	15.9 ± 0.3	15.7 ± 0.2	15.6 ± 0.3	15.7 ± 0.2	15.6 ± 0.2	15.5 ± 0.3	15.6 ± 0.3	15.5 ± 0.3
PCT (%)	0.32 ± 0.02	0.31 ± 0.02	0.35 ± 0.02	0.34 ± 0.02	0.34 ± 0.02	0.34 ± 0.02	0.33 ± 0.01	0.34 ± 0.01	0.33 ± 0.01	0.34 ± 0.01

### Histopathological changes in skin

Histopathological examination of skin samples from intact control rabbits showed preserved architecture of the epidermis, dermis, and hypodermis without detectable pathological changes (Figure 5a). In contrast, skin samples from the infected untreated histopathological subgroup showed focal-to-multifocal epidermal disruption, partial loss of epidermal integrity, and extension of lesions into the dermis (Figure 5b). Affected areas contained serocellular crusts composed of degenerated neutrophils, keratin debris, and basophilic bacterial aggregates (Figure 5c). Hair follicles were reduced in size and number, sebaceous glands appeared atrophic, and band-like lymphocytic infiltration accompanied by moderate dermal edema was observed in the superficial dermis (Figure 5d).

**Table 3:** Descriptive changes in hematological indices of rabbits in the therapeutic group (Group IIa, n = 10) at baseline and after experimental challenge, the first therapeutic vaccination, and the second therapeutic vaccination.

**Hematological parameter**	**Baseline, day 0**	**Challenge, day 1**	**Challenge, day 2**	**Challenge, day 3**	**First therapeutic vaccination, day 1**	**First therapeutic vaccination, day 2**	**First therapeutic vaccination, day 3**	**Second therapeutic vaccination, day 1**	**Second therapeutic vaccination, day 2**	**Second therapeutic vaccination, day 3**
WBC (×10⁹/L)	11.2 ± 0.3	11.4 ± 0.3	11.7 ± 0.3	11.6 ± 0.3	11.4 ± 0.3	11.8 ± 0.3	11.5 ± 0.3	11.5 ± 0.3	11.5 ± 0.3	11.8 ± 0.3
LYM (×10⁹/L)	8.9 ± 0.2	9.0 ± 0.2	9.3 ± 0.2	9.2 ± 0.2	9.0 ± 0.2	9.3 ± 0.2	9.1 ± 0.2	9.1 ± 0.2	9.1 ± 0.2	9.3 ± 0.2
MID/Mon# (×10⁹/L)	0.4 ± 0.1	0.5 ± 0.1	0.6 ± 0.1	0.5 ± 0.1	0.5 ± 0.1	0.6 ± 0.1	0.5 ± 0.1	0.5 ± 0.1	0.5 ± 0.1	0.6 ± 0.1
GRA (×10⁹/L)	2.7 ± 0.1	2.7 ± 0.1	2.9 ± 0.1	2.8 ± 0.1	2.6 ± 0.1	2.9 ± 0.1	2.8 ± 0.1	2.8 ± 0.1	2.8 ± 0.1	2.9 ± 0.1
RBC (×10¹²/L)	8.9 ± 0.2	9.0 ± 0.2	9.1 ± 0.2	9.1 ± 0.2	9.1 ± 0.2	9.1 ± 0.2	9.0 ± 0.2	9.0 ± 0.2	9.0 ± 0.2	9.1 ± 0.2
HGB (g/L)	161 ± 2	162 ± 2	163 ± 2	163 ± 2	163 ± 2	163 ± 2	162 ± 2	162 ± 2	162 ± 2	163 ± 2
HCT (%)	49 ± 1	50 ± 1	51 ± 1	51 ± 0.5	51 ± 1	52 ± 1	50 ± 1	50 ± 1	50 ± 1	52 ± 1
MCV (fL)	69.5 ± 0.5	70.1 ± 0.5	70.2 ± 0.5	70.3 ± 0.5	70.0 ± 0.5	70.5 ± 0.5	70.2 ± 0.5	70.2 ± 0.5	70.2 ± 0.5	70.5 ± 0.5
MCH (pg)	23.3 ± 0.3	23.5 ± 0.3	23.6 ± 0.3	23.5 ± 0.3	23.5 ± 0.3	23.6 ± 0.3	23.6 ± 0.3	23.6 ± 0.3	23.6 ± 0.3	23.6 ± 0.3
MCHC (g/L)	378 ± 3	380 ± 3	382 ± 3	381 ± 3	381 ± 3	382 ± 3	380 ± 3	380 ± 3	380 ± 3	382 ± 3
RDW-CV (%)	–	16.1 ± 0.3	16.2 ± 0.3	16.1 ± 0.3	15.9 ± 0.3	16.2 ± 0.3	15.8 ± 0.3	15.8 ± 0.3	15.8 ± 0.3	16.2 ± 0.3
PLT (×10⁹/L)	635 ± 10	640 ± 10	645 ± 10	645 ± 10	650 ± 10	655 ± 10	642 ± 10	642 ± 10	642 ± 10	655 ± 10
MPV (fL)	4.6 ± 0.2	4.7 ± 0.2	4.8 ± 0.2	4.8 ± 0.2	4.9 ± 0.2	4.8 ± 0.2	4.8 ± 0.2	4.8 ± 0.2	4.8 ± 0.2	4.8 ± 0.2
PDW (%)	15.4 ± 0.3	15.7 ± 0.3	15.6 ± 0.3	15.6 ± 0.3	15.9 ± 0.3	15.7 ± 0.3	15.8 ± 0.3	15.8 ± 0.3	15.8 ± 0.3	15.7 ± 0.3
PCT (%)	0.32 ± 0.03	0.35 ± 0.03	0.34 ± 0.03	0.35 ± 0.03	0.33 ± 0.03	0.34 ± 0.03	0.33 ± 0.03	0.35 ± 0.03	0.34 ± 0.03	0.34 ± 0.03

Values are presented as group means ± standard deviation, with n = 10 rabbits per group. Hematological outcomes were evaluated descriptively because complete individual-level repeated measurements were unavailable for reliable longitudinal inferential analysis. WBC = white blood cell count; LYM = lymphocyte count; MID/Mon# = intermediate or monocyte cell count; GRA = granulocyte count; RBC = red blood cell count; HGB = hemoglobin; HCT = hematocrit; MCV = mean corpuscular volume; MCH = mean corpuscular hemoglobin; MCHC = mean corpuscular hemoglobin concentration; RDW-CV = red blood cell distribution width–coefficient of variation; PLT = platelet count; MPV = mean platelet volume; PDW = platelet distribution width; PCT = plateletcrit.

**Figure 5:**
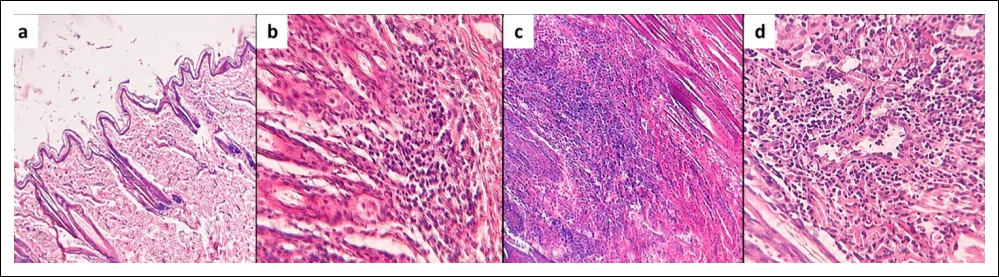
Histopathological changes in the skin of intact control and infected untreated rabbits. Representative hematoxylin and eosin-stained paraffin sections. (a) Preserved architecture of the epidermis, dermis, and hypodermis in an intact control rabbit from Group III (n = 10). (b) Focal-to-multifocal epidermal disruption with partial loss of epidermal integrity and extension into the dermis in an infected untreated rabbit from Group IIb on day 10 post-infection (n = 3). (c) Serocellular crust composed of degenerated neutrophils, keratin debris, and basophilic bacterial aggregates. (d) Reduced size and number of hair follicles, sebaceous gland atrophy, band-like lymphocytic infiltration, and moderate dermal edema. Magnification = ×100 (a) and ×200 (b–d).

### Histopathological changes in lymphoid organs

In intact control rabbits, the spleen showed preserved histological architecture with a clearly defined capsule and trabecular framework and normal differentiation of the red and white pulp (Figures 6a and b). Lymph nodes demonstrated preserved cortical and medullary differentiation, with lymphoid follicles, paracortical areas, medullary cords, and sinuses without detectable pathological changes (Figures 6c and d).

Vaccinated rabbits showed reactive changes in lymphoid tissues. In Group I, regional lymph nodes were enlarged and showed increased numbers of lymphoid follicles with prominent germinal centers, vascular congestion, and reticuloendothelial cell proliferation. The spleen showed mild lymphoid hyperplasia with increased follicular prominence. In Group IIa, lymph nodes were enlarged, edematous, and mildly hyperemic. Histological examination demonstrated pronounced follicular hyperplasia with enlarged germinal centers, whereas the spleen showed increased cellularity and increased numbers of lymphoid and plasma cells. These findings were interpreted as nonspecific reactive lymphoid hyperplasia; their antigen specificity and relationship to vaccine-induced immunity could not be determined histologically (Figure 7).

**Figure 6:**
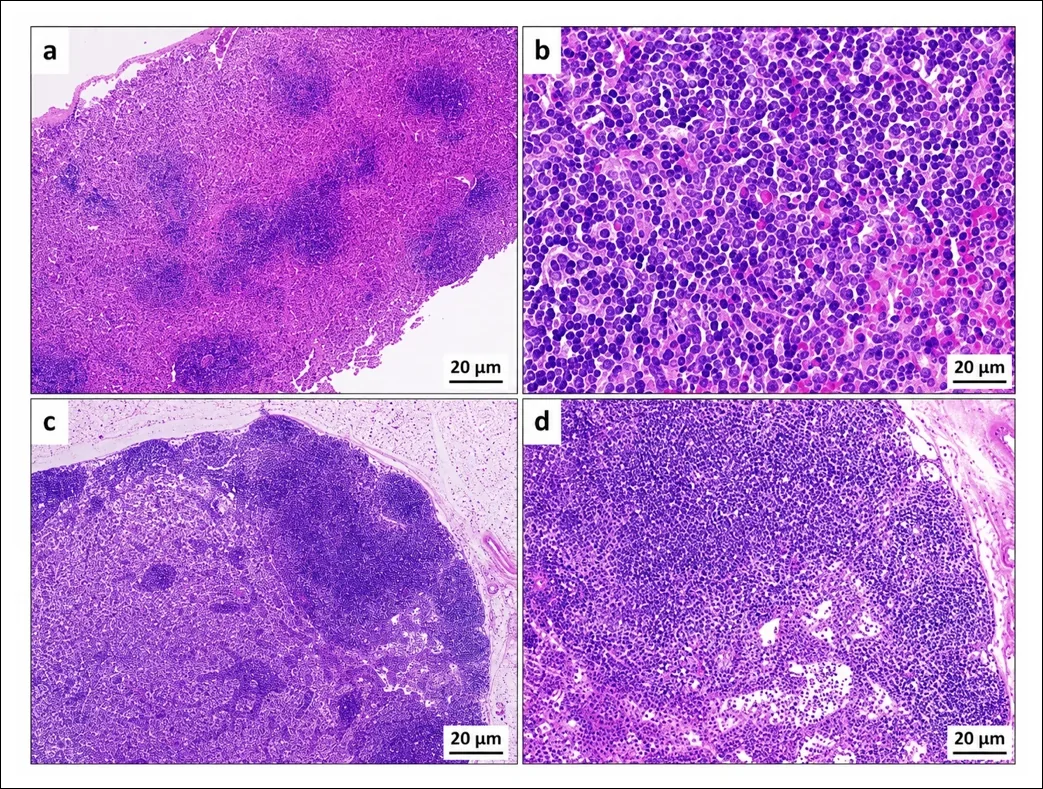
Histological appearance of the spleen and lymph node in intact control rabbits. Representative hematoxylin and eosin-stained paraffin sections from Group III (n = 10). (a) Preserved splenic capsule, trabecular framework, and red- and white pulp architecture. (b) Cellular composition of the splenic tissue, including lymphoid cells, plasma cells, macrophages, and erythrocytes. (c) Preserved cortical and medullary organization of the lymph node. (d) Subcapsular, follicular, and paracortical regions of the lymph node cortex. No detectable pathological changes were observed. Magnification = ×40 (a, c) and ×100 (b, d).

**Figure 7:**
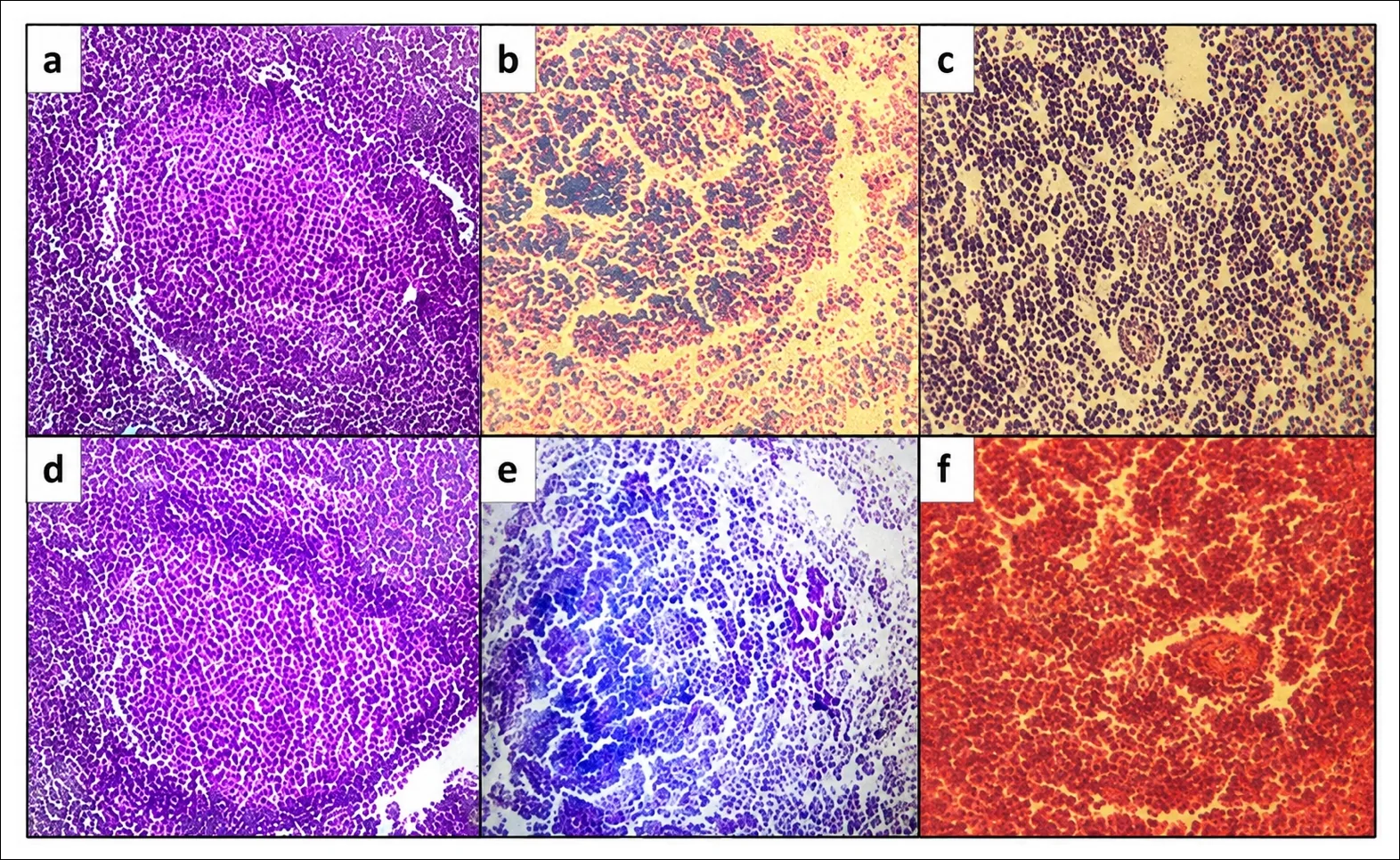
Reactive histopathological changes in the lymphoid organs of vaccinated rabbits. Representative hematoxylin and eosin-stained paraffin sections. (a–c) Prophylactically vaccinated rabbits from Group I (n = 10), showing follicular hyperplasia, prominent germinal centers, vascular congestion, and mild splenic lymphoid hyperplasia. (d–f) Therapeutically vaccinated rabbits from Group IIa (n = 10), showing enlarged lymphoid follicles with prominent germinal centers and increased numbers of lymphoid and plasma cells in the spleen. These findings were interpreted as nonspecific reactive lymphoid changes and do not establish antigen-specific vaccine-induced immunity. Magnification = ×40.

## DISCUSSION

### Prophylactic activity and originality of the bivalent vaccine candidate

To our knowledge, this study provides the first published preclinical evaluation of a live attenuated bivalent vaccine formulation combining *T. verrucosum* with a member of the *T. mentagrophytes* species complex in both prophylactic and post-infection experimental arms within the same rabbit model. The principal prophylactic finding was the absence of clinically visible dermatophytosis lesions in all prophylactically vaccinated rabbits following experimental challenge, whereas visible lesions developed in all animals in the infected untreated histopathological reference group. The difference in lesion incidence was statistically significant (0/10 vs. 3/3; two-sided Fisher’s exact test, p = 0.0035), supporting promising prophylactic activity of the combined vaccine candidate under the conditions of this rabbit model. Nevertheless, this finding should be interpreted cautiously because the untreated reference group included only three animals, clinical lesions were assessed descriptively and without blinding, and quantitative fungal burden assessment was not performed.

The originality of the present study lies in the specific combination of two live attenuated dermatophyte components and the inclusion of both prophylactic and post-infection experimental arms within a single study design. The observed prophylactic activity is broadly consistent with previous evidence that vaccination with live attenuated *T. verrucosum* preparations can help control bovine ringworm [12–14]. Experimental monovalent, bivalent, and polyvalent dermatophyte vaccines have also demonstrated protective activity in cattle and laboratory animal models [15–17]. Most established cattle vaccines are monovalent formulations based on *T. verrucosum*, whereas the present candidate combined *T. verrucosum* with a phenotypically identified member of the *T. mentagrophytes* species complex. However, because monovalent comparator groups were not included, the present study cannot determine whether the *T. mentagrophytes* component provided additional protection or whether the observed prophylactic activity was attributable primarily to the *T. verrucosum* component.

### Epidemiological rationale for the bivalent formulation

The inclusion of a member of the *T. mentagrophytes* species complex was based on broader veterinary evidence indicating that dermatophytes other than *T. verrucosum* may be recovered from cattle and livestock-production environments [2, 3, 9, 11]. Nevertheless, the epidemiological contribution of the *T. mentagrophytes* species complex to bovine dermatophytosis in Kazakhstan remains insufficiently characterized. Recent molecular characterization of 12 cattle-associated *Trichophyton* isolates collected from six geographically separated regions of Kazakhstan confirmed the circulation of cattle-associated dermatophytes and provided an initial regional molecular baseline [8]. However, that investigation was not designed as a prevalence survey and therefore cannot establish the national distribution of individual dermatophyte species.

Standardized epidemiological studies are required to determine animal- and herd-level prevalence, characterize the species and strain composition of circulating dermatophytes, and establish whether a bivalent vaccine formulation is epidemiologically justified under local field conditions. Such data would also help clarify the relative importance of *T. verrucosum* and members of the *T. mentagrophytes* species complex in bovine dermatophytosis in Kazakhstan and provide a stronger epidemiological basis for future vaccine-composition decisions.

### Clinical outcomes following post-infection vaccination

Rabbits vaccinated after experimental infection showed gradual regression of visible crusting and progressive hair regrowth during follow-up. Previous experimental research has suggested that dermatophyte vaccines may exert both prophylactic and post-infection effects in rabbit models [17]. However, the present observations do not demonstrate accelerated recovery or definitive therapeutic efficacy. Group IIb was euthanized on day 10 to provide an early untreated histopathological reference and was not followed throughout the subsequent natural course of infection. In addition, no positive therapeutic comparator receiving a standard antifungal treatment or an authorized commercial vaccine was included.

Consequently, it was not possible to distinguish a vaccine-associated therapeutic effect from spontaneous resolution of experimental dermatophytosis or to compare the candidate formulation with an established therapeutic intervention. Future therapeutic studies should therefore include a longitudinal infected untreated group and an appropriate positive treatment comparator, allowing direct comparison of the timing, rate, and extent of lesion resolution among treatment groups.

### Mycological findings and confirmation of infection

Qualitative mycological culture supported the establishment of viable fungal infection because characteristic dermatophyte growth was recovered from challenged skin material after 5–7 days. However, the study did not quantify fungal recovery as colony-forming units per unit skin area, nor did it subject recovered cultures to species-specific PCR or sequencing. Consequently, the study could not quantify fungal burden, demonstrate complete mycological clearance, independently confirm simultaneous infection with both challenge components, or determine the relative contribution of the two dermatophytes to lesion development.

Future challenge experiments should combine standardized quantitative culture with species-specific molecular identification. Quantitative culture or quantitative PCR would permit more objective assessment of fungal burden and its temporal reduction, whereas species-specific identification would clarify whether both challenge components successfully established infection and whether vaccination affected the two dermatophytes similarly.

### Body weight and rectal temperature responses

Linear mixed-effects analysis identified significant effects of experimental group, day, and the group × day interaction on absolute body weight. However, the prophylactic group had a higher absolute body weight than the therapeutic and intact control groups at baseline. Therefore, between-group differences in absolute body weight cannot be attributed solely to vaccination. The therapeutic group showed a reduction in body weight following experimental infection and partial recovery during follow-up, whereas the prophylactic group showed comparatively limited temporal variation after challenge.

Rectal temperature dynamics also differed significantly among the experimental groups over time. The therapeutic group showed transient elevations in rectal temperature during the acute post-infection period, followed by a gradual return toward baseline values, whereas the prophylactic group showed no sustained temperature elevation after challenge. These physiological observations describe differences in clinical trajectories among the experimental groups but should not be interpreted as independent evidence of vaccine efficacy or systemic safety.

### Hematological observations and safety interpretation

The summarized leukocyte, erythrocyte, and platelet indices showed relatively limited descriptive variation across the recorded sampling points. The available group-level values showed no consistent directional pattern indicating a major hematological disturbance. Nevertheless, complete individual-level repeated measurements were not available for reliable longitudinal or between-group inferential analysis.

Accordingly, the hematological findings cannot establish statistical equivalence among the experimental groups or definitively confirm hematological safety. Future experiments should retain individual animal measurements at each standardized sampling point and apply appropriate longitudinal mixed-effects analyses, with correction for multiple comparisons where applicable. This approach would allow temporal hematological changes to be distinguished more reliably from normal interindividual variation.

### Histopathological findings

Histopathological examination documented epidermal and dermal injury in infected untreated rabbits, including epidermal disruption, serocellular crust formation, reduced size and number of hair follicles, sebaceous gland atrophy, lymphocytic infiltration, and dermal edema. These findings were consistent with active experimental dermatophytosis and broadly aligned with pathological changes reported in experimental rabbit models [17, 18].

Vaccinated rabbits showed follicular hyperplasia, prominent germinal centers, vascular congestion, and increased cellularity in regional lymph nodes and the spleen. These findings were interpreted as nonspecific reactive lymphoid changes. Because histopathological assessment was qualitative, non-blinded, and not supported by predefined grading criteria, morphometric analysis, or fungal-specific staining, the magnitude of local tissue injury and any potential tissue-protective effect could not be formally quantified.

### Immunological interpretation and future immune response studies

This study did not evaluate direct immunogenicity. Antigen-specific antibody titers, cytokine production, lymphocyte proliferation, T-cell responses, and other markers of humoral or cell-mediated immunity were not measured. Although humoral and cellular responses have been discussed in relation to protection induced by dermatophyte vaccines [12, 16], reactive lymphoid hyperplasia alone is not direct evidence of antigen-specific, vaccine-induced immunity.

Therefore, the immune mechanisms and durability of any protection associated with the present formulation remain unknown. Subsequent studies should incorporate predefined humoral and cellular immune endpoints and examine their relationships with standardized clinical-lesion scores, quantitative fungal burden, and mycological clearance. Such measurements would help determine whether clinical protection is associated with measurable antigen-specific immune responses.

### One Health relevance

From a One Health perspective, improved control of bovine dermatophytosis may benefit animal welfare, livestock productivity, farm biosecurity, and occupational health. Cattle-associated dermatophytes can be transmitted to farmers, veterinarians, animal handlers, breeders, and household contacts through direct contact and through contaminated hair, skin scales, equipment, and housing environments [1, 4, 6, 7]. These considerations support integrated surveillance, early veterinary diagnosis, isolation of clinically affected animals, environmental cleaning and disinfection, use of appropriate personal protective equipment, and communication between veterinary and medical services.

However, the present experiment did not assess fungal shedding, environmental contamination, or human exposure. Therefore, any potential effect of the vaccine candidate on environmental transmission or occupational infection remains hypothetical and should not be inferred from the present findings.

### Vaccine quality control and biosafety considerations

Comprehensive characterization of the live vaccine candidate remains necessary. During strain development, the authors used seven serial passages on wort agar to reduce virulence, and the production cultures retained recognizable cultural and microscopic characteristics. However, the authors did not perform comparative residual-virulence testing against the original isolates, genetic stability analysis, or a formal reversion-to-virulence assessment.

Dedicated sterility testing of the final lyophilized preparation, quantitative post-lyophilization viability, batch-to-batch consistency, long-term stability, and validated potency testing were also not conducted. In addition, the study did not evaluate shedding of the attenuated strains, persistence in the environment, or delayed adverse events. These quality control and biosafety parameters require assessment using validated procedures before progressing to target species trials or considering field application.

### Translational relevance of the rabbit model

The rabbit model enabled controlled preliminary assessment of cutaneous challenge, lesion development, physiological changes, and tissue responses. Rabbits are commonly used in experimental dermatophytosis studies because they permit reproducible cutaneous infection and longitudinal observation of lesion development [17, 18]. Nevertheless, rabbits are not the target host for a vaccine intended for bovine trichophytosis.

Differences in skin structure, susceptibility, immune responses, vaccine dose, and natural exposure conditions limit direct extrapolation of the present findings to cattle. The results should therefore be regarded as exploratory preclinical evidence rather than proof of efficacy, therapeutic activity, or safety in the target species.

### Study limitations

The principal limitations of this study were the exploratory sample size and absence of an *a priori* power calculation; the small and early-terminated infected untreated reference group; absence of a longitudinal infected untreated control and positive therapeutic comparator; lack of monovalent vaccine comparator groups; non-blinded clinical and histopathological assessments; absence of validated clinical-lesion and histopathological scoring systems; lack of quantitative fungal burden assessment and species-specific post-challenge confirmation; descriptive hematological analysis; absence of direct immunological measurements; incomplete quality control characterization of the final vaccine preparation; short follow-up; lack of shedding and environmental-persistence studies; incomplete metadata for the challenge cultures; and use of rabbits rather than cattle.

These limitations preclude definitive conclusions regarding therapeutic efficacy, the contribution of each vaccine component, immune mechanisms, long-term safety, and effectiveness under field conditions. They also restrict direct extrapolation of the observed prophylactic activity to the intended bovine target population.

### Future research directions

Overall, the combined live attenuated vaccine candidate showed promising prophylactic activity in the rabbit model, whereas post-infection administration was followed by visible lesion regression and hair regrowth. These findings support further investigation but do not establish definitive therapeutic efficacy or suitability for use in cattle.

The next stage of research should include adequately powered, blinded studies in calves, with longitudinal infected, untreated, and positive treatment controls; monovalent vaccine comparator groups; standardized lesion scoring; quantitative culture or qPCR; species-specific identification; direct immunological assays; validated vaccine quality control testing; shedding assessment; and extended safety monitoring. Target species studies should also evaluate dose optimization, duration of protection, reproducibility across vaccine batches, and performance under controlled exposure conditions before progressing to field evaluation.

## CONCLUSION

The live attenuated bivalent vaccine candidate containing *T. verrucosum* and a member of the *T. mentagrophytes* species complex showed promising prophylactic activity in the rabbit model. No clinically visible dermatophytosis lesions developed in prophylactically vaccinated rabbits after challenge, whereas lesions occurred in all rabbits in the infected untreated histopathological reference group (0/10 vs. 3/3; p = 0.0035). Post-infection vaccination was followed by visible lesion regression and progressive hair regrowth; however, the absence of a longitudinal infected untreated control and a positive therapeutic comparator prevented definitive attribution of clinical recovery to vaccination.

The findings support continued preclinical development of the combined live attenuated formulation and indicate its potential relevance for future control strategies against bovine dermatophytosis. If its prophylactic activity is confirmed in the target species, this approach could improve animal health, reduce disease-associated production losses, strengthen farm biosecurity, and potentially lower occupational exposure to cattle-associated dermatophytes. However, these implications remain prospective because the present study was conducted in rabbits and did not evaluate transmission, environmental shedding, or effectiveness under field conditions.

A major strength of the study was including both prophylactic and post-infection experimental arms within the same rabbit model using a specific bivalent live attenuated formulation. The study also incorporated clinical monitoring, mycological recovery, body weight and rectal temperature assessment, hematological observations, and histopathological examination, providing a multidimensional preliminary evaluation of the vaccine candidate.

Overall, the bivalent live attenuated *Trichophyton* vaccine candidate demonstrated promising prophylactic activity under controlled experimental conditions in rabbits, while the observed post-infection clinical improvement remains preliminary. These findings warrant further investigation but do not establish definitive therapeutic efficacy, long-term safety, or suitability for use in cattle. Robust target species studies are required before the formulation can be considered for practical application in bovine dermatophytosis control.

## DATA AVAILABILITY

The data supporting the findings of this study are presented within the article. Additional study records may be obtained from the corresponding author upon reasonable request, subject to applicable institutional and ethical requirements.

## GENERATIVE AI DECLARATION

The authors declare that no generative artificial intelligence (AI) tools were used in the preparation, writing, editing, or revision of this manuscript.

## AUTHORS’ CONTRIBUTIONS

MU and AS: Conceived and designed the study and supervised the overall project. NB: Isolated the fungal cultures, prepared the vaccine candidate, interpreted the findings, and drafted the manuscript. AN: Interpreted the findings and critically revised the manuscript. NAK, BK, AM, SK, MY, RB, GA, and SI: Participated in the experimental work and data acquisition. MK and NAL: Interpreted the findings. AZ: Interpreted the findings, drafted the manuscript, and critically revised the manuscript. ZZ: Performed the statistical analysis and data visualization. AA: Participated in the experimental work and data acquisition and drafted the manuscript. All authors have read and approved the final manuscript.
